# Transcriptomic analysis to uncover genes affecting cold resistance in the Chinese honey bee (*Apis cerana cerana*)

**DOI:** 10.1371/journal.pone.0179922

**Published:** 2017-06-26

**Authors:** Kai Xu, Qingsheng Niu, Huiting Zhao, Yali Du, Yusuo Jiang

**Affiliations:** 1 College of Animal Science and Technology, Shanxi Agricultural University, Taigu, Shanxi, China; 2 Apiculture Science Institute of Jilin Province, Jilin, Jilin, China; 3 College of Life Science, Shanxi Agricultural University, Taigu, Shanxi, China; University of North Carolina at Greensboro, UNITED STATES

## Abstract

The biological activity and geographical distribution of honey bees is strongly temperature-dependent, due to their ectothermic physiology. In China, the endemic *Apis cerana cerana* exhibits stronger cold hardiness than Western honey bees, making the former species important pollinators of winter-flowering plants. Although studies have examined behavioral and physiological mechanisms underlying cold resistance in bees, data are scarce regarding the exact molecular mechanisms. Here, we investigated gene expression in *A*. *c*. *cerana* under two temperature treatments, using transcriptomic analysis to identify differentially expressed genes (DEGs) and relevant biological processes, respectively. Across the temperature treatments, 501 DEGs were identified. A gene ontology analysis showed that DEGs were enriched in pathways related to sugar and amino acid biosynthesis and metabolism, as well as calcium ion channel activity. Additionally, heat shock proteins, zinc finger proteins, and serine/threonine-protein kinases were differentially expressed between the two treatments. The results of this study provide a general digital expression profile of thermoregulation genes responding to cold hardiness in *A*. *c*. *cerana*. Our data should prove valuable for future research on cold tolerance mechanisms in insects, and may be beneficial in breeding efforts to improve bee hardiness.

## Introduction

Temperature is one of the most important abiotic factors influencing insect activity and geographic distribution [1]. Insects may face daily or seasonal temperature fluctuations in temperate climates and subzero temperatures in frigid zones, severely compromising metabolic and physiological activity [2]. Although differences in population distribution patterns [3–5], developmental stages [6] and diapause status [7] all contribute to the considerable variation in cold tolerance among insects, they have evolved a series of consistent physiological and behavioral strategies to mitigate cold-related damage. Behaviorally, some insects (e.g., monarch butterflies *Danaus plexippus*) perform long-distance migration to warm habitats [8], while others take shelter in thermally buffered microclimates, such as tree bark crevices or under snow and frozen soils [1, 9]. Physiologically, insects are broadly divided into freeze-tolerant and freeze-susceptible species, based on their adaptation mechanisms; the former group can survive after a percentage of their extracellular fluids freeze, whereas the latter group cannot [10]. Winter survival rates and cold resistance is improved in freeze-susceptible insects through the regulation of physiological and metabolic pathways that lower their supercooling point (SCP), the temperature below which insects will freeze [11, 12]. The SCP is thus an indicator of cold resistance capacity. Insect SCP can be altered through extreme dehydration, purging the gut of ice nucleators, and producing multimolar concentrations of cryoprotectants [13, 14].

Cryoprotectants are divided into high molecular weight antifreeze-associated proteins and small-molecule cryoprotectants. The former group includes heat shock proteins (HSPs), antifreeze proteins, antifreeze glycolipids, and other cold-induced stress proteins, which all exhibit distinctive structures and properties that are critical to overcoming low temperatures [15–17]. In insects, the synthesis of small cryoprotectants is substantially common; these compounds include polyhydric alcohols, sugars, and glycerol [18]. Studies on cold-shock and chilling tolerance in *Drosophila* have shown that energetic reserves (e.g., glycogen, triacylglycerols, and proline) may be important in cold-coping mechanisms [19]. In the cold-hardy gall fly (*Eurosta solidaginis*), for example, the larvae exhibit high levels of glycerin, sorbitol, glucose, and trehalose biosynthesis [20]. Other overwintering insects also synthesize glycoproteins and various amino acids; these molecules likely play a key role in lowering the SCP as part of a cold-stress response [21, 22].

The vast majority of research on insect cold resistance has focused on solitary species, with social insects being relatively ignored. The honey bee (*Apis* spp.) is an eusocial insect and major pollinator, with high economic value worldwide; an understanding of cold-tolerance mechanisms in the honey bee thus has important practical implications. Honey bees have been anthropogenically introduced to a wide range of tropical and temperate geographical regions. Physiological and behavioral thermoregulation is essential for the ectothermic honey bees at both individual and colony levels [23, 24]. Bee colonies tend to have high stable temperatures in the central core area (where the brood is located) under a variety of environmental conditions [25], achieved via the clustering and layering of worker bees to form an insulating shell [23, 26]. Because of this ability to maintain a constant temperature, studies on thermoregulation in honey bees tend to focus on the colony. However, individual forager bees, for example, must still survive colder environmental conditions in early spring and late autumn to successfully gather food [27]. Currently, we know that honey bees maintain their body temperature above ambient conditions through burning fuel during flight [27]. However, when temperatures drop below 12°C, individual honeybees are unable to fly, and they become comatose at temperatures below 10°C [28]. More work is required to understand the molecular regulatory mechanisms of cold resistance in honey bees.

The Chinese honey bee (*Apis cerana cerana*) is an important endemic species that is critical to environmental improvement and ecosystem balance in China [29]. *A*. *c*. *cerana* has a lower SCP [30] and is more cold-hardy than the Western honey bee (*A*. *mellifera*), displaying high forager activity below 14°C and pollinating winter-flowering plants in southern China [31]. Thus, *A*. *c*. *cerana* should be an effective model organism for studying transcriptional regulation of cold resistance in honey bees. In this study, we characterized gene expression in cold-treated *A*. *c cerana* adults, using Illumina sequencing technology to examine their transcriptomes. Through identifying key temperature-regulated genes and gene networks, this research should offer important insight into the mechanisms of cold tolerance.

## Materials and methods

### Insect treatments

Chinese honey bees (*A*. *c*. *cerana*) were maintained in an experimental apiary from the College of Animal Science and Veterinary Medicine of Shanxi Agriculture University (Taigu, China). Winter bees were bred on September 15, 2015 and emerged on October 5, 2015. Newly emerged adults were marked with paint. Adults 20-day post-emergence were collected from three hives on October 25, 2015, when the environmental temperature in this region ranged from 6°C to 15°C. Three hundred adults (n = 100/hive) were collected and each individual bee was placed into 15 mL centrifuge tubes with small pores. Bees were then divided into two groups (n = 150/group) and treated for 2 h at either 0°C (cold treatment; ZOT) or 25°C (normal-temperature treatment; ZRT) in constant-temperature incubators. After 2 h, 60 randomly selected bees (n = 30/group) were allowed to recover for 30 min before mortality was calculated as an indication of phenotypic (cold-tolerance) variation. The remaining individuals (120/group) were immediately frozen in liquid nitrogen for total RNA extraction and RNA-seq analysis.

### Library construction

Total RNA was extracted using TRIzol reagent (Ambion, Foster City, CA, USA) following the manufacturer’s protocol. Total RNA concentration was determined using a Qubit® RNA Assay Kit and the Qubit® 2.0 Fluorometer (Life Technologies, Carlsbad, CA, USA), and RNA sample quality was assessed with agarose gel electrophoresis. Novogene Bioinformatics Technology Co., Ltd, Beijing, China (http://www.novogene.cn/) performed RNA library construction. Sequencing libraries were generated using the NEBNext® Ultra™ Directional RNA Library Prep Kit for Illumina® (NEB) in accordance with the manufacturer’s protocol. After clustering index-coded samples, each library preparation was sequenced on an Illumina HiSeq 2000 platform and 50-bp single-end (SE) reads were generated.

### Read mapping to the reference genome

Raw reads in the fastq format were first processed using in-house Perl scripts. they were then cleaned by removing of low-quality reads and those containing adapters or poly-N (quality limit 0.05). High-quality clean reads were used for downstream analyses. The Q20, Q30, and GC contents of the clean data were calculated. Next, clean tags were retained and mapped to the reference genome of *Apis cerana* (https://www.ncbi.nlm.nih.gov/genome/?term=Apis+cerana). TopHat was the selected as the mapping tool because it can generate a splice junction database based on the gene-model annotation file, leading to better mapping results than other non-splice-site mapping tools.

### Bioinformatic analysis of RNA-seq data

Reads mapped to each gene were counted in HTSeq (version 0.5.4p3) [32]. Fragments per kilobase of transcript per million fragments sequenced (FPKM) of each gene were calculated from gene length and mapped read count [33]. Differential expression analysis of the two temperature treatments was performed using the DEGSeq R package v1.12.0 [34]. The Benjamini and Hochberg method was used to adjust P-values [35]. The significance threshold for differential expression was set at a corrected P-value < 0.05 and log2 (fold-change) of 0.5.

### GO and KEGG pathway enrichment analyses

Gene ontology (GO) enrichment analysis of the differentially expressed genes (DEGs) was performed using the GOseq R package (version 1.10.0), with a correction for gene length bias. GO terms with a corrected P <0.05 were considered significantly enriched by DEGs. Significant enrichment (corrected P < 0.05) in a Kyoto Encyclopedia of Genes and Genomes (KEGG) pathway analysis was tested with KOBAS (http://kobas.cbi.pku.edu.cn/).

### qRT-PCR analyses

Differential gene expression was verified with qRT-PCR (quantitative reverse transcription polymerase chain reaction). First-strand cDNA was synthesized from 1 μg of total RNA, using the ReverTra Ace-a First-strand cDNA Synthesis Kit (TaKaRa, Shiga Prefecture, Japan). Primers designed for each gene are given in S1 Table. The qRT-PCR thermocyling program was as follows: 95°C for 30 s; followed by 45 cycles of 95°C for 5 s and 60°C for 30 s. The *β-actin* gene was used as an internal standard for normalization. All samples were performed in triplicate. The results were normalized to the expression level of the constitutive Lbr. Relative quantification of PCR results was determined using the 22DDCt method [36].

## Results

### Differential phenotypes of ZOT and ZRT

In present study, we subjected two groups of bees to different temperatures (0°C and 25°C) for 2 h. Comparisons of activities over time revealed that physiological function of the 0°C group declined gradually, whereas it did not in the 25°C group. After 20 min, the honeybees under 0°C went into cold coma (abdominal stop peristalsis). At the end of different temperature treatments, we found no mortality in either group (n = 30/group).

### Mapping of RNA-seq reads to the *A*. *cerana* genome

The three 0°C libraries (ZOT-1, ZOT-2, and ZOT-3) and three 25°C libraries (ZRT-1, ZRT-2, and ZRT-3) generated 41.56–59.70 million (M) raw reads per sample (Table 1). After quality control, the two groups averaged 44 667 109 and 49 380 011 clean reads, respectively. All short reads in both groups mapped perfectly to the *A*. *cerana* genome, and 70.91–73.92% of the tags in all six libraries could be uniquely mapped to the reference genome. Approximately 45% of reads were mapped to known exons and 27% were located in predicted intergenic or intronic regions.

**Table 1:** Statistics for filtering and mapping reads.

Sample name	ZOT-1	ZOT-2	ZOT-3	ZRT-1	ZRT-2	ZRT-3
**Raw reads**	49154366	49126550	41564122	59697374	48085270	46703842
**Clean reads**	47036956	47103902	39860468	57267848	46088490	44783694
**Q20(%)**	96.12	96.48	96.24	96.29	96.21	96.34
**Q30(%)**	91.01	91.77	91.29	91.42	91.25	91.5
**GC content(%)**	39.1	38.98	39.35	38.51	38.47	39.1
**Total mapped**	34957172 (74.32%)	34662679 (73.59%)	29541499 (74.11%)	43093870 (75.25%)	33408430 (72.49%)	33647776 (75.13%)
**Multiple mapped**	624038 (1.33%)	682230 (1.45%)	544638 (1.37%)	941355 (1.64%)	728148 (1.58%)	631477 (1.41%)
**Uniquely mapped**	34333134 (72.99%)	33980449 (72.14%)	28996861 (72.75%)	42152515 (73.61%)	32680282 (70.91%)	33016299 (73.72%)
**Non-splice reads**	21399542 (45.5%)	20872449 (44.31%)	18213648 (45.69%)	26360258 (46.03%)	20035849 (43.47%)	20806494 (46.46%)
**Splice reads**	12933592 (27.5%)	13108000 (27.83%)	10783213 (27.05%)	15792257 (27.58%)	12644433 (27.44%)	12209805 (27.26%)

### Analysis of DEGs

**Fig 1:**
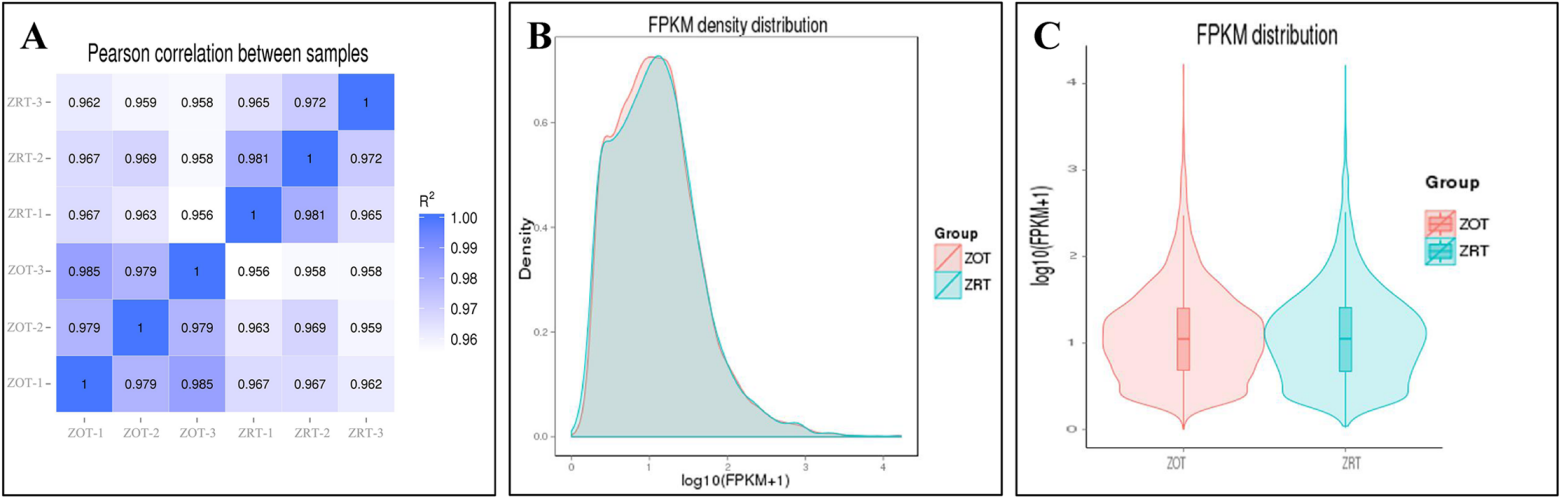
Bioinformatic analyses of RNA-seq data. (A) Pearson correlation between samples. (B) FPKM density distribution of two groups. (C) FPKM distribution of two groups. ZOT: 0°C treatment; ZRT: 25°C treatment.

Before DEG analysis, an RNA-seq correlation test (Pearson’s) and FPKM distribution were used to check sample variation across the two treatments. Strong positive correlations (R^2^ > 0.8; Fig 1A) were found between samples, indicating reasonable sample selection. The FPKM and density distributions showed that most genes were in the same groups (Fig 1B and 1C).

**Fig 2:**
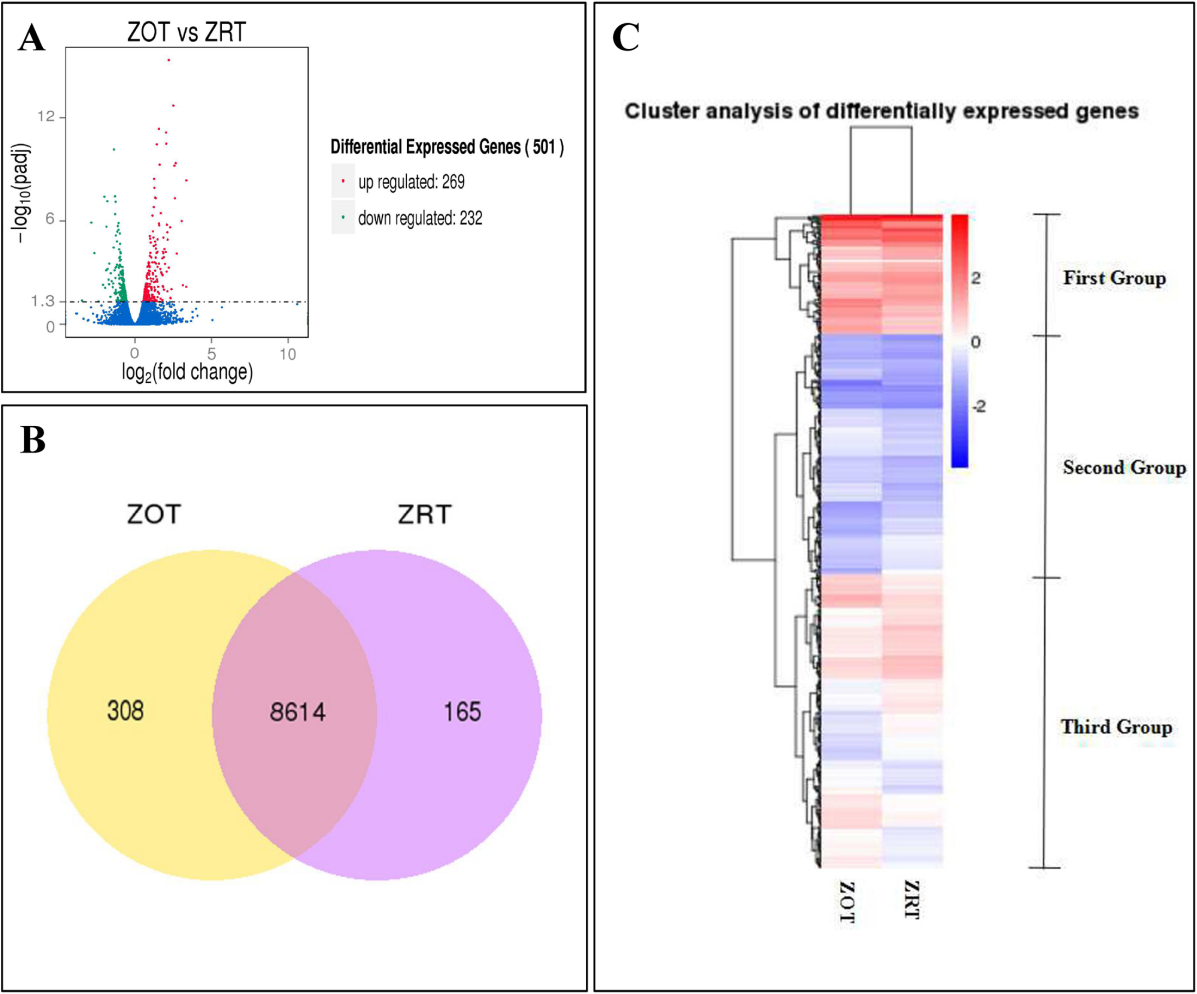
Bioinformatic analyses of DEGs (differentially expressed genes). (A) DEGs distribution of the two treatment groups (ZOT: 0°C, ZRT: 25°C). (B) Venn diagrams showing the number of genes expressed in the two groups. (C) Cluster analysis of DEGs.

Based on FPKM values, we detected 10 651 genes in the *A*. *cerana* genome. To better decipher the mechanisms driving the response to low temperatures, the DESeq tool was used to calculate differential expression. A loose transcript detected with p-adjusted < 0.05 and log2^FoldChange^ was used to compare the ZOT library to the ZRT library (S2 Table). The ZOT group contained 269 significantly upregulated DEGs and 232 significantly downregulated DEGs (Fig 2A). The 501 DEGs and annotations found across the two groups are shown in S3 Table. Venn diagram analysis revealed that 8614 genes overlapped between the two groups, while 308 genes were expressed only in ZOT and 165 were expressed only in ZRT. Cluster analysis showed that DEGs between ZOT and ZRT were divided into three groups: upregulated in the first, downregulated in the second, and with varied expression in the third (Fig 2C).

Gene annotation identified differential expression between the two treatments included nine genes encoding heat shock proteins (HSPs), seven encoding serine/threonine-protein kinase genes (STKs), and twelve encoding zinc finger protein (ZFPs) (S4 Table).

### GO enrichment analysis of DEGs

**Fig 3:**
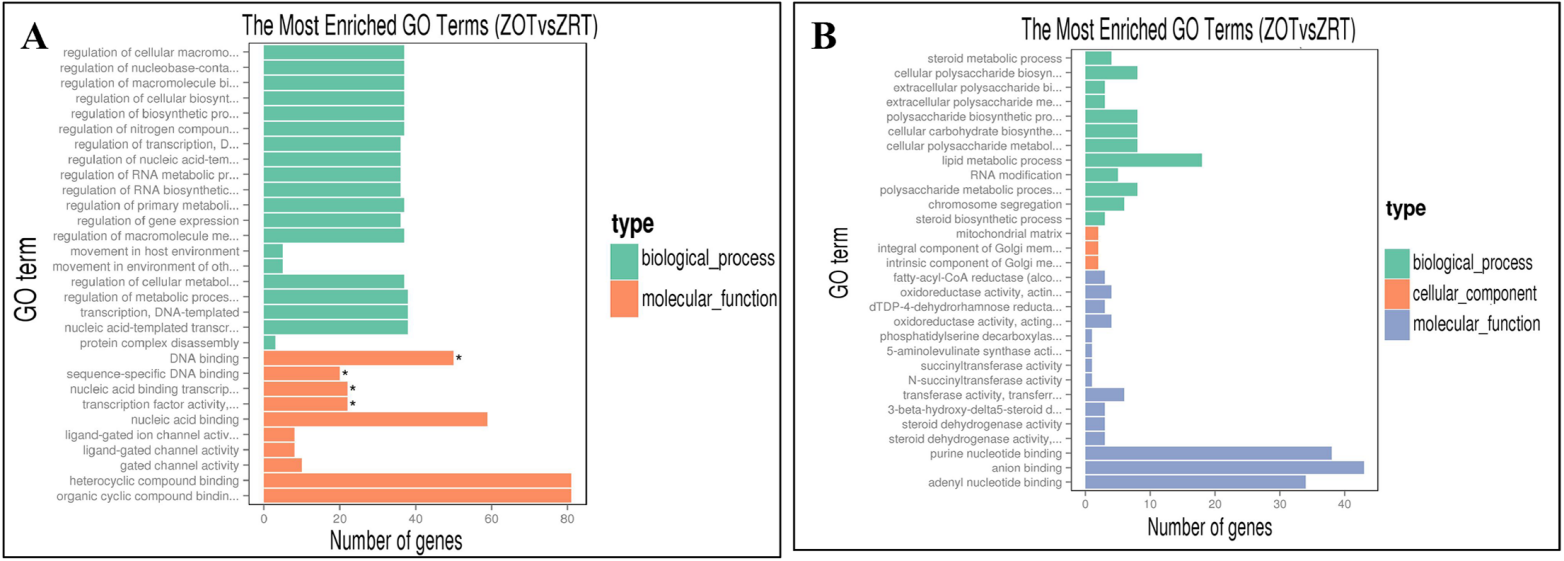
Bar graph showing gene ontology (GO) of DEGs between ZOT (0°C) and ZRT (25°C). (A) The most enriched GO terms for upregulated DEGs between the two treatment groups. (B) The most enriched GO terms for downregulated DEGs between the two treatment groups.

The GOseq tool was used for GO analysis of DEGs, based on the Wallenius non-central hyper-geometric distribution [37]. Upregulated DEGs mostly enriched DNA binding, gated channel activity, as well as biosynthesis and metabolism regulation (Fig 3A). Down-regulated DEGs were mostly involved in anion binding, oxidoreductase activity, mitochondrial matrix, lipid metabolism, polysaccharide biosynthesis, and steroid metabolic processes (Fig 3B). More cold-tolerance-related GO terms were associated with upregulated than down-regulated DEGs. These cold-tolerance-related terms were associated with biological processes (energy biosynthesis and metabolism [of glycogen, nucleic acids, amino acids, polyol]; stimulus response, movement, ion transport, signal transduction), molecular function (binding; activities of gate channels, calcium channels, kinases, transmembrane transporters), and cellular components (microtubule cytoskeleton, cell, cell part, protein complex).

### KEGG analysis of DEGs

KEGG pathway enrichment analysis [38] of DEGs revealed that these transcripts were assigned to 63 reference pathways (S5 Table). Among them, metabolic pathways were the most enriched. These DEGs enriched glycine, serine, and threonine metabolism; carbon metabolism; purine metabolism; glycerophospholipid metabolism; inositol phosphate metabolism; fatty acid metabolism; alanine, aspartate, and glutamate metabolism; amino sugar and nucleotide sugar metabolism; as well as cysteine and methionine metabolism. Additionally, many enriched pathways were involved in signal transduction pathways related to cell differentiation and proliferation, repair of injured organs, as well as regulation of cytosolic calcium concentration (e.g., Wnt signaling pathway, Hippo signaling pathway–fly, FOXO signaling pathway, Hedgehog signaling pathway, and phosphatidylinositol signaling system) [39, 40].

### Validation of RNA-seq by qRT-PCR

To validate the expression profiles identified from RNA-seq, we performed qRT-PCR on several cold-stress-related DEGs (nine HSPs, seven STKs, and twelve ZFPs). The comparative analysis showed that qRT-PCR-detected expression patterns supported RNA-seq results, with only one DEG (in the STK protein family) being inconsistent (Table 2). However, the fold change of expression patterns from RNA-seq and qRT-PCR still deviated slightly, probably due to methodological differences [41].

**Table 2:** Results of qRT-PCR and RNA-seq on genes encoding cold-resistance proteins.

Protein family	Gene name	log2^FoldChange^		Protein family	Gene name	log2^FoldChange^	
		RNA-seq	qRT-PCR			RNA-seq	qRT-PCR
**HSPs**	*HSC70-3*	-0.93	-2.29	**STKs**	*PLK1*	-0.78	-1.17
**HSPs**	*HSC70-4*	-1.79	-2.12	**STKs**	*MKNK1*	0.66	0.23
**HSPs**	*HSP10*	-1.03	-1.07	**ZFPs**	*ZBED1*	-2.65	-2.99
**HSPs**	*HSP60*	-0.81	-1.19	**ZFPs**	*ZFP-25*	0.78	0.32
**HSPs**	*HSP90*	-1.45	-2.7	**ZFPs**	*ZFP-36*	1.21	0.67
**HSPs**	*sHSP22*.*6*	-1.68	-2.22	**ZFPs**	*ZFP431*	1.56	0.6
**HSPs**	*sHSP24*.*2*	-2.85	-3.49	**ZFPs**	*ZFP582*	-0.9	-0.18
**HSPs**	*HSF2BP*	2.61	1.49	**ZFPs**	*ZFP708*	0.64	0.39
**HSPs**	*HSF5*	1.13	0.81	**ZFPs**	*ZFPN*	0.81	0.4
**STKs**	*CG31145*	0.54	-0.3	**ZFPs**	*ZFPR*	1.14	0.61
**STKs**	*STYX*	-0.73	-1.35	**ZFPs**	*Zkscan5*	0.76	0.12
**STKs**	*STKA2*	0.79	0.39	**ZFPs**	*ZMPN13*	-1.28	-1.63
**STKs**	*Mig15*	-0.82	-0.49	**ZFPs**	*ZIP13*	-0.86	-0.59
**STKs**	*PAKm*	0.63	0.23	**ZFPs**	*ZIP9*	-0.68	-1.39

## Discussion

Insects vary considerably in their ability to survive low temperatures [14]. Insect susceptibility to physiological experimentation increases the difficulty of exploring cold-resistance mechanisms in the honey bee. However, with the availability of the Eastern honey bee (*A*. *cerana*) whole genome [42] and the development of RNA-seq, we now have effective alternatives for studying the molecular mechanisms of cold resistance.

This study identified DEGs between honey bees treated under two temperature conditions. Our data are in line with existing research on other insects. Previously, a DNA microarray study in *Drosophila melanogaster* identified 36 transcripts associated with low-temperature treatments (0°C for 2 h) [18]. Furthermore, an RNA-seq study in New Zealand stick insects showed that genes expressed under cold exposure (-5°C for 1 h) included HSPs, cuticular genes, as well as others involved in carbohydrate metabolism and physiological variation [43]. Transcriptomic and proteomic analysis in Chinese white wax scale insects (*Ericerus pela*) from different climatic regions found 2386 DEGs, involved in energy metabolism, catalytic activity, and response to stimuli [13].

To further investigate the molecular mechanism of cold resistance in *A*. *c*. *cerana*, we used RNA-seq to compare DEG transcript levels. We demonstrated that the number of upregulated DEGs (269) was slightly higher than the number of downregulated DEGs (232) between the two treatment groups. Next, KEGG analysis revealed that metabolic pathways (including carbon, amino acid, and nucleic acid metabolism) were the most enriched. Thus, energy metabolism was critical to cold resistance in honey bees, in line with the observation that honey bees consume honey and pollen to produce heat during low temperatures [23, 26]. Data from physiological studies of various insects [20, 44] also support these results. Finally, signal transduction pathways are important in cold stress responses [45], and in this study, four such pathways were found to be enriched.

Based on GO analysis, the DEGs were most enriched in biological processes, and specifically in metabolic processes. As the main energy sources for honey bees are glucose, fructose, maltose, and sucrose, it was unsurprising that the detected DEGs were involved in glucose metabolism. These included *mitogen-activated protein kinase-binding protein 1* (*MAPKBP1*), *poly(ADP-ribose) glycohydrolase* (*PARG*), *UDP-glucuronosyltransferase 1–8* (*UTG 1–8*), and *maltase A3* (*Mal-A3*). *MAPKBP1* is a key transcription factor in the NF-κB signaling pathway [46]. The role of insects *MAPKBP1*remains unclear, but a previous study found that endogenous *MAPKBP1* and scaffold protein genes co-localize to stress granules; thus, the expression of this gene might be related to cold tolerance in honey bees [47]. *RAPG* is a hydrolyzing enzyme and regulator of poly(ADP-ribose) activity, producing free ADP-ribose residues to affect several important biological functions, such as DNA repair, chromosome stability, and maintenance of normal cell function [48]. Like other *UTG1* members, *UTG 1–8* is involved in glucuronidation, a process that enhances the conversion of lipophilic xenobiotics and endobiotics to more water-soluble compounds. For example, UTG 1–8 can split UDP-glucuronic acid and catalyze glucuronic acid transfer to a small hydrophobic molecule [49]. Finally, *Mal-A3* is a maltase that catalyzes maltose hydrolysis to glucose [50].

Free amino acids have a strong affinity to small molecular compounds, leading to increased cell fluid concentration, as well as improved cell water retention and stability [1]. Indeed, high accumulation of free amino acids in the hemolymph appears to be a major biochemical characteristic of insect cold-stress response [20–22]. Corroborating those findings, we identified DEGs associated with serine, lysine, glycine, threonine, and methionine metabolism. Two upregulated and zero downregulated genes were linked to methionine and threonine, while four downregulated and zero upregulated genes were linked to serine, lysine, and glycine. These results suggest that methionine and threonine may act as cryoprotectants in honey bees. Interestingly, although lipids are another major energy source, lipid metabolism was most strongly enriched in downregulated DEGs in this study. Lipids are an essential energy source in cell membrane synthesis, and energy from lipid metabolism is primarily allocated to long-distance travel in insects [51]. Therefore, lipid metabolism may be downregulated in the cold because honey bee flight ability is hampered. However, lipids may power honey bees to flight at the appropriate temperature, while maintaining membrane structure in the cold.

A major biological signal to cold response is variation in intracellular calcium ion concentration [52]. Upregulated GO terms related to calcium channel activity included *voltage-dependent calcium channel subunit alpha-2/delta-3* (*CACNA2D3*) and *small conductance calcium*-*activated potassium channel protein* (*SK*). *CACNA2D3* is an auxiliary member of the alpha-2/delta subunit family in the voltage-dependent calcium channel complex; it regulates calcium ion influx upon membrane polarization [53]. *SKs* are gated solely through an increase in internal calcium concentration and are therefore extremely sensitive to calcium. Our observation of *SK* and *CACNA2D3* expression indicated that greater calcium ion activity and increased intracellular calcium concentration improves cold tolerance in honey bees. Our results are similar to previous findings in *D*. *melanogaster* [52].

The DEGs related to metabolic processes and the calcium channel activity terms identified indicate that some small cryoprotectants (including glucose, methionine, threonine and calcium) may play an important role in the cold-coping mechanisms of the honey bee. We can apply the information gained from our study to optimize honey bee diet in the winter. Currently, the most important supplement for honey bees in the winter in most China northern states is sucrose (processed white table sugar), while the addition of free amino acids and minerals in diets has often been overlooked. The results of this study highlight the importance and necessity of dietary supplementation with small cryoprotectants. The partial substitution of invert sugar (its main component sugars are glucose and fructose) for sucrose and extra methionine, threonine and calcium added in the production of stored, it could improve overwintering success and decrease the loss of honey bees.

Antifreeze-associated proteins, unlike small-molecule cryoprotectants, can induce ideal antifreeze effects through catalyzing specific biochemical reaction and affecting the conformation of the resulting molecules [54–56]. In this research, some DEGs were linked to three such proteins (HSPs, STKs, and ZFPs) and can be considered candidate genes for subsequent studies on honey bee cold-tolerance mechanisms. Additionally, based on these candidate genes, we can generate the corresponding proteins *in vitro* as a protectant against further cold stress.

HSPs are a super family of chaperone proteins rapidly biosynthesized in response to various environmental stress factors [57, 58]. We found nine DEGs associated with HSPs: two upregulated (*heat shock factor 2-binding protein* [*HSF2BP*] and *heat shock factor protein 5* [*HSF5*]) and seven downregulated (*heat shock protein 10 kDa* [*HSP10*], *heat shock protein sHSP24*.*2a* [*sHSP24*.*2a*], *heat shock protein sHSP22*.*6* [*sHSP22*.*6*], *heat shock protein 60 kDa* [*HSP60*], *heat shock protein 90 kDa* [*HSP90*], *heat shock 70 kDa protein cognate 3* [*HSC 70–3*], *heat shock 70 kDa protein cognate 4* [*HSC 70–4*]). As in other insects, the expression of *HSPs* can be induced quickly with low temperatures. Recently, a study in *Sitodiplosis mosellana* suggested that *HSP90* and *HSP70* expression at low temperatures (-5°C) was higher than under normal conditions [59]. Further, rapid cold hardening significantly upregulated three *HSP* genes (*hsp40*, *hsp23*, and *hsp10*) in *Folsomia candida* [60]. However, after prolonged cold exposure, *HSPs* expression levels would drop [61], indicating that the resultant state of the honey bees in the 0°C treatment was not induced by any new stimuli, and that a self-defense mechanism may have been established.

Unlike typical HSPs, *HSC70* function varies across multiple species and treatments. Some studies suggest that *HSC70* is expressed constitutively and thus, not stress-inducible [62]. Others report that *HSC70* is involved in thermoregulation, as well as protein synthesis, folding, transport, and degradation [63]. Our results for *HSC70* indicated that its expression is also sensitive to low temperature. Additionally, *HSFs* mediate *HSPs* induction through binding to the heat shock element of *HSP* genes, explaining why *HSFs* mRNA levels clearly increased in adult fruit flies exposed to 4°C [64]. *HSF5* is part of a protein family that modulates *HSPs* expression; therefore, it is likely related to thermoregulation. Indeed, *HSF5* expression was significantly higher in winter than in summer [65]. Further, *HSF2BP* primarily modulates *HSF2* activation; an increase in its transcripts can be induced following a heat-shock treatment, but it also appears to be the most insensitive to temperature change among the *HSP*s [66]. Overall, our results show that lower temperatures downregulated *HSPs* and upregulated *HSFs* in honey bees, similar to findings in *D*. *melanogaster* [18]. These outcomes suggest that *HSPs* expression and activity varies in response to cold stress.

The zinc finger protein family (so-called due to the presence of the zinc finger domain) is large and widely distributed across taxa (plants, animals, microorganisms) [54]. These proteins are involved in growth and development as well as in abiotic stress responses (e.g., to salt, water, heat, and cold) [67, 68]. Recently, three differentially upregulated *ZFPs* were identified in heat-treated silkworm eggs [41]. This study also identified twelve *ZFPs*, including seven upregulated genes and five downregulated genes. Our results together with those of previous studies demonstrate that *ZFPs* are important in the cold-stress response of insects and that high expression of some specific *ZFPs* improves cold tolerance. Additionally, we found seven DEGs related to *STKs*, a major protein kinase group [69] that is implicated in modulating abiotic stress responses (including the cold) [70, 71]. However, research on insect *STKs* and *ZFPs* has been limited. Thus, our current results and their implications for honey bees should be considered with caution. The roles of differentially expressed *STK* and *ZFP* genes in the molecular mechanisms underlying honey bee cold-stress response warrant further study.

## Conclusions

In conclusion, 501 DEGs were identified across two temperature treatments. The results of GO analysis revealed that DEGs significantly enriched terms associated with sugar and amino acid biosynthesis and metabolism, as well as with calcium ion channel activity. Various temperature-sensitive proteins (e.g., HSPs, STKs, and ZFPs) were also differentially expressed across temperature treatments. Our study provides insight into the molecular mechanisms of cold resistance in *A*. *c*. *cerana* and offers a scientific foundation for exploring candidate genes linked to cold stress in social insects.

## Supporting information

S1 Table Primers used for qRT-PCR analysis on differentially expressed genes (DEGs). (DOC)

S2 Table DEGs between ZOT and ZRT, based on corrected P values and log2^FoldChange^. (XLS)

S3 Table Annotation of DEGs in NR, NT, and SwissProt databases. (XLS)

S4 Table Annotation of HSPs, STKs, and ZFPs. (XLS)

S5 Table The most enriched KEGG pathway for DEGs between ZOT and ZRT. (XLS)
