# Correction: Transcriptomic analysis to uncover genes affecting cold resistance in the Chinese honey bee (*Apis cerana cerana*)

**DOI:** 10.1371/journal.pone.0184468

**Published:** 2017-08-31

**Authors:** Kai Xu, Qingsheng Niu, Huiting Zhao, Yali Du, Yusuo Jiang

In the Funding section, the grant number from the funder National Nature Science Foundation of China is listed incorrectly. The correct grant number is: 31372386.
