# Supplementary material for: Transcriptomic analysis to uncover genes affecting cold resistance in the Chinese honey bee (Apis cerana cerana)
Source: PLoS One. 2017 Jun 26;12(6):e0179922. doi: 10.1371/journal.pone.0179922 (PMC5484514; doi:10.1371/journal.pone.0179922)
Supplement: S1 Table — (DOC) [file pone.0179922.s001.doc]

**S1Table. Primers used for qRT-PCR analysis on differentially expressed genes (DEGs).**

**Primers of** Heat shock proteins (HSPs)

| Primer name | GenBank ID | Forward primer (5**'**-3**'**) | Reverse primer (5**'**-3**'**) |
| --- | --- | --- | --- |
| *HSC70-3* | 107997049 | ACCAGCACCACGAGGAATAC | CATCAGGTGTAAGGCGGTTT |
| *HSC70-4* | 107996313 | GAATCCGTTTGCAATCCTGT | TTCTTCGATGGTTGGTCCTC |
| *HSF2BP* | 107996546 | ACGGAAGTTTCGTCGCATT | CGTCCCTCAGGTGTAGCAGA |
| *HSP10* | 108000341 | TTCTTGTGCAAAGAGCTGAAG | TTTGTCCAGGTCCAATTGCT |
| *HSP60* | 108000480 | TGCGTACGGCACTTACTGAC | CCATTGGCATTTGAGGTTCT |
| *HSP90* | 408928 | AGGTCAGTTTGGTGTAGGTTT | TCTCCATTGTCAGGGCGAAC |
| *HSF5* | 107994746 | TCTTTCGTTGCGATTTCCGC | CCCGCGTCGAGATACTGTTC |
| *sHSP22.6* | KF150018.1 | ATCACCGAGCCGATTGGA | TGTCACTACGAAACAGTTCAGCC |
| *sHSP24.2* | KF150019.1 | GGGAGAGGATTTGAAGACACC | AACAGCGATGAGGTCTTAGCA |

Primers of Heat shock proteins (STKs)

| Primer name | GenBank ID | Forward primer (5**'**-3**'**) | Reverse primer (5**'**-3**'**) |
| --- | --- | --- | --- |
| *CG31145* | 107998746 | GCCGGTAATATCTGCTTCCA | TCGCGACGAAAGATTTATCC |
| *STYX* | 107993674 | GGTCTTTCCCAAACATGTGC | CTCAATTGCGCCATGAAAC |
| *STKA2* | 108004128 | CAGAATTGATGCCACCACCT | CTCATCATCAGTTGCATTTGG |
| *Mig15* | 108003038 | CCTTGATGGTGTGTTCATGC | CACTTCCGCCTTTAGCTGAC |
| *PAKm* | 108003675 | TTTGTCTTCGGGAAATCTGG | GCCCGAAATTGTTCATGAGT |
| *PLK1* | 107999607 | TTCCGTGCAACTATCATCCA | TAGTGCCAGCGGTTGTAGTG |
| *MKNK1* | 108000369 | GAAAGGTATCGCTCACAGGG | AGCCAAGATCAAAATCGCAC |

Primers of Heat shock proteins (STKs)

| Primer name | GenBank ID | Forward primer (5**'**-3**'**) | Reverse primer (5**'**-3**'**) |
| --- | --- | --- | --- |
| *ZBED1* | 108004414 | AAGATACACCCGATCGTTGG | GTCAGGGATGGTACGTGCTT |
| *ZFP-25* | 107998072 | TAAACCATTTGCGTGCCATC | TTTATATGGACGGGCTCCTG |
| *ZFP-36* | 410758 | CCCAATCCTTACGTTCAAGC | CCGGTAGAACCGTGTAAGGA |
| *ZFP431* | 108001041 | AGTTTGCGCTTGCATTCTCG | TTTTACCGCACTCGGGACAT |
| *ZFP582* | 108002579 | AAAGTTTGCGCTTGCATTCT | CTCGGGACATGTGAATGTTG |
| *ZFP708* | 107997300 | GAACGCATTCACACAGGAGA | CTGCATGAACCCATCGAAC |
| *ZFPN* | 108002202 | GCCCAGACTTGCAGTCAAAT | TGTTGTTGCTCGAACCTTTG |
| *ZFPR* | 107998742 | AGGCAACAGACACCTCAACC | TGGTTGAGAGGGTTCAACGG |
| *Zkscan5* | 107993647 | AAACAGACTCGCCAAAGACC | CCGGAAATGCCAAATTACAT |
| *ZMPN13* | 107994936 | CAGATTTGAACGGGACGAGT | ACGCTGCCATAATCGTATCC |
| *ZIP13* | 410710 | GCATCCCATTTGTTGAATCC | CTGTGGGTGGTTCTTTCCTC |
| *ZIP9* | 107995542 | CTGCTCCTTGTCTTGCTCTTG | AGCATTTTCGGCACTTGGTA |

**Primers of House keeping gene (*β-actin***)

| Primer name | Forward primer (5**'**-3**'**) | Reverse primer (5**'**-3**'**) |
| --- | --- | --- |
| *β-actin* | ACTACGGCCGAACGTGAAAT | GGAAAAGAGCCTCGGGACAA |
